# Influence of Jamaican Cultural and Religious Beliefs on Adherence to Pharmacotherapy for Non-Communicable Diseases: A Pharmacovigilance Perspective

**DOI:** 10.3389/fphar.2022.858947

**Published:** 2022-03-14

**Authors:** Robyn Brown, Caryl James Bateman, Maxine Gossell-Williams

**Affiliations:** ^1^ Department of Sociology, Psychology, and Social Work, Faculty of Social Sciences, The University of the West Indies, Mona Campus, Kingston, Jamaica; ^2^ Section of Pharmacology and Pharmacy, Faculty of Medical Sciences, The University of the West Indies, Mona Campus, Kingston, Jamaica

**Keywords:** adherence, pharmacotherapy beliefs, cultural, religious, pharmacovigilance

## Abstract

Worldwide, socio-cultural determinants have been shown to influence the beliefs of patients about their health and decision making for treatment. This is consistent with the evidence that cultural and religious beliefs affect illness conceptualization and behaviors of Jamaican patients living with non-communicable diseases, such as diabetes mellitus and hypertension. Despite these known socio-cultural influences, an acknowledgment of relevance of adherence to pharmacotherapy has been grossly understudied. Furthermore, while poor adherence to pharmacotherapy, especially in the management of patients living with non-communicable diseases is associated with adverse drug reactions; reporting of such information in the pharmacovigilance process is inadequate. We review previous studies on the cultural and religious beliefs within the Jamaican context that may contribute to poor adherence to pharmacotherapy, especially among those patients living with non-communicable diseases. We support the ongoing perspective that current pharmacovigilance processes need retooling with the inclusion of socio-cultural influences on adherence to pharmacotherapy.

## Introduction

Non-communicable diseases (NCDs) represent a significant portion of the global disease burden and are managed by lifelong pharmacotherapy. According to the World Health Organization (WHO), NCDs disproportionally affect developing countries and among these patients, adherence to pharmacotherapy rates is less than fifty percent (Sabaté, 2003). These challenges coupled with the limited resources to care for the health needs of its population increases the morbidity and mortality rates of NCDs in developing countries (Rose et al., 2016). Developing countries, such as Jamaica, encounter unique challenges at the various levels of the healthcare system (Figueroa et al., 1999; Wilks et al., 2001; Gossell-Williams et al., 2014; Hartzler et al., 2014; Mitchell-Fearon et al., 2015; Wilson et al., 2018). However, arguably and more important in impacting public health is having a health-conscious populace that is motivated to leverage the health system to the betterment of their overall well-being.

According to terminology agreed by European consensus, adherence to pharmacotherapy is defined as the process by which patients take their medications as prescribed (Vrijens et al., 2012). A major contributor to adherence to pharmacotherapy is culture which is defined as a decision-making heuristic that can be found in values, beliefs, or social norms (Nunn, 2012). More specifically, various sociocultural factors have been found to affect patient health behaviours, decision-making and health outcomes (Oates et al., 2020), of which multidimensional factors, such as cultural and religious beliefs are among the most recognized (Leporini et al., 2014). Hordern (2016) emphasized the need for physicians to understand and respect the significance of cultural and religious beliefs of patients, as they inform the manner of their engagement with recommended treatment.

The goal of this paper is to explore how these socio-cultural factors impact adherence to pharmacotherapy for common NCDs in Jamaica, as well as to highlight their potential role in the pharmacovigilance process. We highlight peer reviewed publications reporting adherence to pharmacotherapy in Jamaican patients with NCDs after 2003, the year of implementation of the National Health Fund, which subsidizes the cost of drugs to Jamaicans (https://www.nhf.org.jm/images/pdfs/nhf_act_merged.pdf). The available studies involving Jamaican patients with NCDs suggest their adherence to pharmacotherapy is poor, with concerns about adverse drug reactions, having to take the drugs consistently even when no symptoms are being experienced and the general inconvenience featuring prominently. (Table 1) (Duff et al., 2006; Chambers et al., 2008; Pusey-Murray et al., 2010; Mowatt, 2013; Gossell-Williams et al., 2014; Welsh et al., 2015; Bridgelal-Nagassar et al., 2016; Wilson et al., 2018; Adeniyi et al., 2021; Barrett-Brown et al., 2021).

**TABLE 1:** Overview of the studies presenting measures of non-adherences among Jamaicans living with NCDs. For all studies the measure of adherence.

References	Chronic disorder of patients (sample size)	Non-adherence rate (based on self-report)	Theme identified by patients as reasons for non-adherence
Adeniyi et al. (2021)	DM and HTN (85)	40% for patients with only DM	Financial difficulty, Insurance problems, medication non-availability at local pharmacy and difficulty collecting medication
		31.2% for patients with only HTN	
		30.8% for both conditions	
Barrett-Brown et al. (2021)	DM (101)	53.5%	High pill burden—“tired of taking medications”
Bridgelal-Nagassar et al. (2016)	DM (260)	33% overall; 28.5% amongst those with health-insurance	Not reported
Chambers et al. (2008)	Systemic lupus erythematosus (75)	44%	Financial difficulty, medication non-availability in local pharmacy, fear of side effects, preference for herbal therapies, perception of mild disease, religious beliefs
Duff et al. (2006)	DM (133)	55%	Not reported
Gossell-Williams et al. (2014)	52 HTN (52)	Range from 9.6 to 40.4%	Adverse drug reactions, prefer not to take medication in the absence of symptoms, general inconvenience and pill burden
Mowatt (2013)	DM (104)	34%	Not specified
Pusey-Murray et al. (2010)	Mental disorders (344)	55.3%	Side-effects, running out of medication, forgetting to take medication, medication makes things worse
Watson and Ferrillo, (2021)	DM and HTN (116)	19.2% for patients with DM	Did not recognize the importance of taking medication consistently to manage chronic illness
		32.1% for patients with HTN	
Welsh et al. (2015)	HTN (48)	56.3%	Adverse drug reactions, pill burden, difficulty obtaining medication at the pharmacy, preference for herbal therapies
Wilson et al. (2018)	HTN (307)	17.5% for patients with uncontrolled HTN	Not reported
		21.4% for patients with controlled HTN	

DM, diabetes mellitus; HTN, hypertension.

### Beliefs About Illness

Beliefs as defined by Macionis (2015, p. 95) are “specific thoughts or ideas that people hold to be true”. Understanding patients’ health beliefs is imperative as research has consistently shown that this predicts their health behaviors, including adherence to pharmacotherapy (Street and Haidet, 2011). Cultural beliefs relate to what is internalized to inform thinking and actions, while religious beliefs relate to beliefs in a social institution based on recognizing the sacred (Macionis, 2015). These terms are not mutually exclusive, as religion may be considered a cultural system (Bonney, 2004).

The way illness is conceptualized through a particular cultural lens has a direct impact on the kinds of actions taken in its management. Considering the diversity implicit within the Jamaican experience, it is difficult to define a cultural attitude towards pharmacological intervention which is all encompassing. However, insights have been offered to explain relevant ethnographic factors. Mitchell (1983) gave a comprehensive description of the ethnomedical landscape in Jamaica during the 1980s which is still relevant nearly 40 years later. The way in which Jamaicans conceptualize and treat diseases involve an emphasis on symptomatology and bodily-feeling*;* there is a need to feel a “cure” working in the body to counteract a particular ill-effect and the elimination of symptoms through treatment signifies the elimination of the disease, regardless of whether it is chronic and incurable (Mitchell, 1983). These perceptions have been corroborated in survey studies done amongst patients in Jamaica and the Jamaican diaspora living in the United Kingdom and the United States of America where poor adherence to pharmacotherapy was related to the practice of “leaving-off” pills, which was justified by the idea that illness exists only in the presence of symptoms (Myfanwy and Watkins, 1988; Brown et al., 2007; Alhomoud et al., 2013; Smith, 2012; Gossell-Williams et al., 2014; Welsh et al., 2015).

### Beliefs About Alternative Treatments

The desire to feel a cure working is one potential explanation for the cultural beliefs in herbal preparations observed throughout Jamaican society. Many herbal preparations, especially in the form of teas, elicit instantaneous bodily sensations, such as “warmth” or “bitterness.” For example, Cerasee tea (made from the plant *Momordica charantia*) is viewed as an effective treatment for diabetes mellitus because of the perceived counteracting or balancing effect of the bitter taste (Mitchell, 1983; Smith, 2012). It is important to explore not only whether herbal preparations are efficacious, but also the cultural cognitions that lead Jamaican’s to combat illness in a particular way. Sobo (1993) examined the health perspectives of impoverished rural Jamaicans and how they approach well-being. There was an emphasis on natural, easily accessible options with attention being paid most carefully to the balancing of the system through “washouts” and “purification” rather than taking prescription drugs.

Considering Jamaica’s extensive history of traditional knowledge systems (Payne-Jackson and Alleyne, 2004; Seaga, 2005; Delgoda et al., 2010; Picking et al., 2019; Adeniyi et al., 2021; Bateman James, 2021), it is not surprising that Jamaicans from various backgrounds turn regularly to alternative treatments. Records of the 1866 Commission investigating the Morant Bay Rebellion highlight the long history of Jamaicans consulting physicians on current medicine, but then relying on alternative treatments (Barima, 2016). Payne-Jackson and Alleyne (2004) in their 1991–1992 questionnaire-based survey of rural communities in Jamaica highlighted the involvement of physicians and alternative practitioners (e.g., bush-doctors, spiritual mothers and occult healers) in the treatment of illness, which inevitably leads to several treatment recommendations being used sequentially and simultaneously. This approach towards managing illness remains popular and thus the prescribed pharmacotherapy is often replaced or supplemented with alternative treatment options, popularly believed to be effective (Mitchell, 1983; Gardner et al., 2000; Delgoda et al., 2004; Gossell-Williams et al., 2008; Delgoda et al., 2010; Picking et al., 2011; Welsh et al., 2015; Foster et al., 2017; Owusu et al., 2020; Adeniyi et al., 2021). Patient testimonials about the anti-cancer benefits of herbal preparations travel by word of mouth, reaffirming their perceived efficacy and swaying others towards their utilization. Owusu et al. (2020) found in a western Jamaican sample of patients with hypertension and type two diabetes mellitus that the motivation for an alternative treatment plan included the belief that it is the more natural choice, and the belief that it is beneficial to utilize pharmacological and herbal preparations concurrently.

In exploring the factors which contribute to the popularity of herbal preparations in Jamaica, especially for the treatment of cancer, Foster et al. (2017) found that 80% of patients surveyed used them, often without the knowledge of their oncologist. In a survey canvasing the level of herbal preparation use concomitantly with pharmacological interventions amongst Jamaicans in both urban and rural settings, Picking et al. (2011) found that 72.6% of respondents had used herbal preparations in the last 12 months. Additionally, of those who were using both forms of treatment simultaneously, only 19.4% shared this information with their physician (Picking et al., 2011) corroborating the findings of Foster et al. (2017). One reason cited for withholding the self-administering of herbal preparations is that the physician simply did not ask (Picking et al., 2011; Smith 2012; Owusu et al., 2020). This presents a major concern about the risk of adverse drug reactions to concomitant users and reasons for this gap in patient-physician communication need to be explored.

### Religious Beliefs About Illness and Pharmacotherapy

Several religions are practiced in Jamaica and although the influence of religious beliefs on illness and healing perception have been reviewed (Sutherland et al., 2014), the relationship between religious beliefs and adherence to pharmacotherapy has been minimally explored. At an individual level, the influence of religion is highly subjective with some patients interpreting their healthcare provider’s recommendations as God’s way of helping them to help themselves, while others take a more fatalistic approach believing that their illness is God’s will and therefore how it unfolds is out of their hands (Brown et al., 2007; Shahin et al., 2019; Smith, 2012; Hope et al., 2020). Taking this line of reasoning one step further, some highly religious patients may believe that their illness is a punishment from God for improper religious adherence and although they may respect their physician’s diagnosis and recommendations, their ultimate concern rests with the judgement of their higher power (Rumun, 2014).

Chambers et al. (2008) explored the influence of Christianity among Jamaicans living with systemic lupus erythematosus; they found that several of the patients held a “strong belief in the possibility that they could be healed of lupus at any time” p.767. Amongst this cohort however these beliefs did not appear to hinder their adherence to pharmacotherapy. Anecdotal reports included the belief that healing was obtained at a crusade, leading to a patient ceasing to take their medication and their eventual death. Another patient reported finding prayer to be an effective way for them to relieve some of their pain and discomfort until they were able to replenish their medication (Chambers et al., 2008).

### A Role for Cultural and Religious Beliefs in the Pharmacovigilance Process

Jamaica, as a full member of the World Health Organization global database since 2012, collects individual case safety reports of adverse drug reactions following guidelines established by the International Conference on Harmonization (E2B (R3) Individual case Safety Report Specification and Related Files; https://www.ich.org/page/e2br3-individual-case-safety-report-icsr-specification-and-related-files). This global pharmacovigilance database facilitates detection of possible adverse drug reactions; however, the processes of data analysis and whether there is adequate focus on patient safety has been questioned (Edwards, 2017; Streefland, 2018; Ibrahim et al., 2021). According to Edwards (2017, p. 365) “We must develop a more holistic evaluation of suboptimal therapeutic outcomes, and do this without apportioning unnecessary blame”.

Recent systematic reviews and observational studies highlight the relevance of socio-cultural determinants on adherence to pharmacotherapy among patients living with NCDs (Dhar et al., 2017; Niriayo et al., 2018; Swihart et al., 2018; Shahin et al., 2019; Al-Ganmi et al., 2020; Park et al., 2020; Raza et al., 2020; Świątoniowska-Lonc et al., 2021); however, the dearth of published literature suggests such awareness in pharmacovigilance processes is yet to be realized. Although poor adherence to pharmacotherapy is a common predictor of adverse drug reactions; the converse is also true (Gellad et al., 2011; Adem et al., 2021; Elangwe et al., 2020) and therefore enriching pharmacovigilance processes with what is referred to as ‘the patient’s voice’ may facilitate causality analysis of adverse drug reactions (Simon et al., 2020).

## Conclusion

Cultural and religious beliefs have been shown to negatively impact adherence to pharmacotherapy in patients living with NCDs; despite this, current pharmacovigilance processes fail to give these beliefs much consideration in understanding poor adherence to pharmacotherapy, a known contributor to adverse drug reaction occurrences. Elaborating on the possible relationship between these factors and poor adherence to pharmacotherapy in the Jamaican context was the goal of this article, with several notable themes emerging. Among these were the cultural perception of illness as existing only in the presence of symptoms, and the importance of combating illness by targeting symptoms with treatments that one can feel acting in the body. Also featured were religious influences on the way patients make meaning of their experience of illness, along with the role they ascribe to physicians in facilitating their treatment.

Based on the empirical articles reviewed, the cultural and religious beliefs of Jamaicans about pharmacotherapy may be a significant contributor to poor adherence rates among patients living with NCDs. Adding this information is important in a comprehensive pharmacovigilance process, and future research should explore the optimization of adverse drug reaction reporting with patient data in these domains.

## Data Availability

The original contributions presented in the study are included in the article/Supplementary Materials, further inquiries can be directed to the corresponding author.
